# Effect of maternal oxygen supplementation for parturient undergoing elective cesarean section by high-flow nasal oxygen compared with room air on fetal acidemia: study protocol for a randomized controlled trial

**DOI:** 10.1186/s13063-024-07927-y

**Published:** 2024-01-22

**Authors:** Yun-Hui Li, Gui-Yu Lei, Jun Guo, Meng Yi, Yu-Jing Fu, Gu-Yan Wang

**Affiliations:** 1 Department of Anesthesiology, Beijing Tongren Hospital, Capital Medical University, Beijing, 100730 China; 2 Department of Gynecologic and Obstetric, Beijing Tongren Hospital, Capital Medical University, Beijing, 100730 China

**Keywords:** Oxygen, High-flow nasal oxygen, Fetal acidemia, Umbilical arterial lactate, Intrauterine resuscitation, Cesarean section

## Abstract

**Background:**

Maternal oxygen supplementation is usually used as an intrauterine resuscitation technique to prevent fetal hypoxia and acidemia during delivery. However, there has been a great deal of controversy regarding the effects of prophylactic maternal oxygen during cesarean section, during which the incidence of fetal acidemia seems to be higher compared with that during labor. High-flow nasal oxygen (HFNO) can improve oxygenation better in patients with high-flow oxygen airflow. The purpose of this study is to determine whether maternal oxygen supplementation with HFNO has a positive effect on fetal acidemia during cesarean section through umbilical arterial blood gas analysis.

**Method:**

This prospective, single-center, randomized, double-blinded trial will enroll 120 patients undergoing cesarean section. Participants will be randomly assigned to the HFNO group or air group at a 1:1 ratio. For parturients in the HFNO group, the flow rate is 40L/min, and the oxygen is heated to 37℃ with humidity 100% oxygen concentration through the Optiflow high-flow nasal oxygen system. And for the air group, the flow rate is 2 L/min with an air pattern through the same device. The primary outcome was umbilical artery (UA) lactate. Secondary outcomes include UA pH, PO_2_, PCO_2_, BE, the incidence of pH < 7.20 and pH < 7.10, Apgar scores at 1 and 5 min, and neonatal adverse outcomes.

**Discussion:**

Our study is the first trial investigating whether maternal oxygen supplementation with HFNO can reduce the umbilical artery lactate levels and the incidence of fetal acidemia in cesarean section under combined spinal-epidural anesthesia.

**Trial registration:**

ClinicalTrials.gov NCT05921955. Registered on 27 June 2023.

**Supplementary Information:**

The online version contains supplementary material available at 10.1186/s13063-024-07927-y.

## Introduction

Maternal oxygen supplementation has been widely used for intrauterine resuscitation intervention in the clinic to prevent neonatal acidemia. Previous studies have reported that maternal hyperoxygenation has a positive effect on fetal heart rate pattern during the second stage of labor in the presence of suspected fetal distress [1], and is associated with a low overall rate of the composite neonatal adverse outcome [2].

However, fetal acidemia is more likely to occur during a cesarean section than during labor. The functional residual capacity and reserve oxygen capacity of pregnant women are decreased by uterine migration and obesity, and these changes are worsened in the supine position. Moreover, maternal oxygen consumption is increased by fetal and placental metabolism. In addition, spinal block-induced sympathicolysis decreases maternal systolic arterial blood pressure, causing hypoxia and anaerobic metabolism in infants [3]. Previous study suggests that the indication of cesarean section is also a risk factor of fetal acidemia [4].

Studies focused on the effect of maternal oxygen supplementation during cesarean section have shown mixed results. Prior researches demonstrated that maternal oxygen improved PaO_2_ in the umbilical artery (UA) [5–8]. A prospective trial observed higher UA pH values in the receiving oxygen group [9]. Moreover, a recent meta-analysis specifically assessing the use of supplemental oxygenation at the time of cesarean delivery concluded that oxygen supplementation was associated with lower rates of UA pH less than 7.2 [10]. But, several trials have shown that maternal oxygenation had no effect on UA pH [8, 11, 12] and fetal wellbeing [13].

In the previous trials, the concentrations and flow of oxygen inhalation were inconsistent. The maximum oxygen flow rate was not more than 10 L/min [7]. It has been demonstrated that providing 35% oxygen via a facemask does not significantly improve fetal oxygen delivery during cesarean section under spinal anesthesia [14]. But 60% supplementary oxygen increased fetal oxygenation [6, 8]. Therefore, the different concentrations and flows may have different effects.

The high-flow nasal oxygen (HFNO) has been widely used in clinical practice, which provides humidified warm oxygen at a high flow rate, thus generating low positive airway pressure, and improving oxygenation and comfort [15]. HFNO has been safely used in pre-oxygenation of general anesthesia for women in labor [16, 17], providing superior oxygenation during rapid sequence induction compared to standard facemask [17]. However, most of those studies focused on maternal effects, few studies evaluated fetal indicators as the primary outcome.

The aim of this study was to investigate the immediate fetal effects of supplemental oxygenation with HFNO by measuring umbilical arterial blood gas values at the time of term, elective cesarean section. We hypothesized that such supplementation would have a positive effect on umbilical artery lactate levels, a marker of metabolic acidosis and neonatal morbidity.

## Method

### Study design

This trial is a parallel, prospective, single-center, randomized controlled trial. Using a difference test, we focus on the effect of maternal supplemental oxygenation with HFNO on the fetus compared with room air. The study will be performed at Beijing Tongren Hospital, Capital Medical University. And we will adhere to the Good Clinical Practice guidelines and the Declaration of Helsinki during the entire study. The protocol of this trial was approved by the Clinical Research Ethics Committee of Beijing Tongren Hospital on March 9, 2023, with the latest vision 3.0 (approval NO.TREC2023-KY021). This trial has been registered on ClinicalTrials.gov (identify: NCT05921955). Any important protocol modifications will be submitted to the ethical committee for approval. The SPIRIT 2013 checklist is listed in additional file 1.

### Participants

**Fig. 1:**
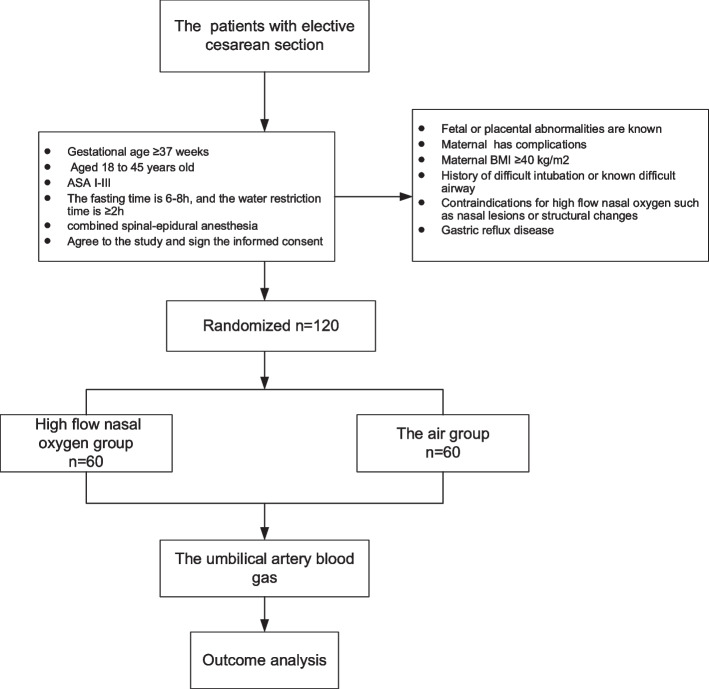
Flowchart of the study

We will recruit 120 patients scheduled to undergo elective cesarean section in the Beijing Tongren Hospital. One trained researcher will screen the eligible patients, explain all the details, and sign the informed consent before the day of surgery. The flowchart of the study is illustrated in Fig. 1, and the SPIRIT figure of enrollment, interventions, and assessments is presented in Table 1.

**Table 1:** Standardized Protocol Items: Recommendations for Interventional Trials (SPIRIT) schedule for enrolment, intervention, and assessment

	Study period				
	Enrollment	Allocation	Post-allocation		
	The day before the surgery	The surgery day	End of injection to delivery	Baby born	Discharge
Enrollment					
Enrollment	X				
Informed consent	X				
Allocation		X			
Intervention					
Intervention			X		
Assessments					
Maternal Information	X	X	X	X	X
Intraoperative situation			X		
UA blood gas analysis				X	
Newborn outcome				X	X

### Inclusion criteria

Participants who meet all the following criteria will be enrolled:

1. An elective cesarean section at Beijing Tongren Hospital
2. Gestational age ≥ 37 weeks
3. Aged 18 to 45 years old
4. American Society of aAnesthesiologists (ASA) classification I–III
5. Patients fasted for 6–8 h, and the water restriction time was ≥ 2 h.
6. Anesthesia: combined spinal-epidural anesthesia
7. Agree to the study and sign the informed consent

### Exclusion criteria

Participants will be excluded if they have any of the following conditions:

1. Fetal or placental abnormalities are known(premature rupture of membranes, severe intrauterine distress, and fetal malformation)
2. Maternal with complications (eclampsia or cardiovascular or cerebrovascular disease)
3. Maternal BMI ≥ 40 kg/m.^2^
4. Difficult intubation history or known difficult airway
5. Contraindications for HFNO such as nasal lesions or structural changes
6. Gastroesophageal reflux disease

### Randomization and blinding

Randomization will be conducted by a computer-generated blocked randomization sequence. All participants conforming to the inclusion criteria are randomly assigned to the HFNO group or the air group at a ratio of 1:1. And the random numbers are placed in opaque envelopes and arranged in serial numbers, which are kept by an anesthesia technician. On the day of surgery, the envelope will be opened by the anesthesia technician when the patients enter the room and the grouping result can be obtained. Others are unaware of the group information.

Unblinding will only be allowed when serious adverse effects occur. Participants will be distributed and assigned in numerical order. The outcome will be assented by assessors, and the data will be calculated by statisticians who will not participate in the treatment. All original records including informed consent and CRF together with related letters will be reserved for 10 years and then destroyed according to the hospital standards.

### Intervention

Participants will be monitored with electrocardiograph (ECG), pulse oxygen saturation, and non-invasive blood pressure after entering the room. Heart rate (HR), systolic blood pressure, and diastolic blood pressure were recorded every minute in the supine position. A 22G intravenous cannula will be inserted in the dorsum of a hand. All the patients will receive an intravenous 500 ml of Ringer’s lactate solution.

Combined spinal-epidural anesthesia will be performed at the L2–3 or L3–4 with the patient in the right lateral position. The anesthesiologist determined the local anesthetic dose of ropivacaine (12–15 mg) according to the height of the parturient. After injection of the local anesthetic solution, a 19G terminal closed epidural catheter will be placed. At the same time, oxygen will be supplied to patients in the HFNO group. The flow rate of 40 L/min heated to 37℃ with humidity 100% oxygen concentration is delivered through the Optiflow high-flow nasal cannula system. And the patients of the air group receive the 2 L/min with air pattern through the same device.

The patient is immediately placed in the supine position once the anesthesia procedure is completed with a left lateral table tilt of 15°. The upper level of the sensory block will be assessed in the midline, using a pin to test for the absence of touch sensation.

HR and blood pressure will be recorded every minute following the injection of the spinal drug until the baby is delivered. The vasopressor bolus is administered as soon as the hypotension is occurred. If hypotension is not corrected after 1 min, then additional boluses will be administered every minute until hypotension is corrected (HR ≥ 75 bpm, 50 μg of phenylephrine boluses; HR < 75, 6 mg of ephedrine boluses).

Umbilical arterial and venous blood samples will be obtained for blood gas analysis from a segment of the umbilical cord double-clamped before the baby’s first breath.

### Outcome assessment

The primary outcome is UA lactate levels. The secondary outcomes include UA pH, PO_2_, PCO_2_, BE, the incidence of pH < 7.20 and pH < 7.10, Apgar scores at 1 and 5 min, and the neonatal adverse outcomes in the operating room and ward (such as intubation, mechanical ventilation, and admission to neonatal unit). Others include the number of hypotensive episodes, the number of vasopressor boluses required, the incidence of tachycardia, bradycardia, arrhythmias, the time from subarachnoid injection to skin incision, the time of oxygen supplementation, and the time from skin incision to fetal delivery.

### Sample size calculation

According to a retrospective observational study, there was a strong inverse correlation between lactate and pH (*r* =  − 0.77, *p* < 0.001) [18], and the clinically significant change of UA pH is assumed to be 0.02 pH units [19]. According to the experience of our hospital, the mean of the UA lactate is 2.0 (0.028) mmol/l. With a probability of *α* = 0.05, *β* = 0.2, and power of 0.80, the sample size was 53 according to a 2-side 2-sample equal-variance *t* test. Considering 15% patient loss, we are going to recruit 120 patients. The sample size was calculated with PASS software.

### Statistical methods

Data analysis will be performed according to the modified intention-to-treat population. Moreover, the per-protocol analysis will be used as a sensitivity analysis. We expect very few patients to be lost. As a result, the missing data will be minimized when analyzing primary outcomes. Multiple imputation will be used to handle missing data if needed.

Shapiro–Wilk test is used to test normality. Quantitative indicator is expressed as mean ± standard deviation or median (interquartile range, IQR), while qualitative data is expressed as median (interquartile, IQR). Quantitative indicators are tested by independent sample *t* test or Mann–Whitney *U* test according to data distribution. Qualitative data is tested by the chi-square test/Fisher’s exact test. Statistical analysis will be performed by SPSS 27.0.

### Safety consideration

Participants can withdraw at any time during the trial. We will keep strictly to the indications and contraindications to minimize the risk of adverse events (AEs) which might be caused by the high-flow nasal oxygen. In the process of clinical research, all AEs will be recorded form truthfully and in detail, including the clinical features, occurrence time, severity, duration, related treatment, and outcomes of AEs. When an AE occurs, it is associated with high-flow nasal oxygen, then we will stop using the device. The severe adverse events will be reported to the Clinical Research Ethics Committee as soon as possible. And we will be responsible for the subsequent treatment.

### Oversight and monitoring

An anesthesiologist, an obstetrician, and a nurse midwife were employed at the coordination center for this study. A statistician will guide the statistical analysis of the data obtained for this study. The data monitoring committee (DMC) consists of an anesthesiologist, an ethics expert, a statistician, and a clinical trial management expert. All team members are independent of the sponsor, and there are no conflicts of interest. The DMC will hold a monthly meeting to review research progress, check the integrity of the data, and monitor the occurrence of AEs.

### Data collection and management

There is one researcher to record all the data of the preoperative, intraoperative, and postoperative and collect the case report forms timely, completely, and correctly. And another researcher will check the data to ensure data accuracy and productivity level. All data are confidential all the time. The research records and data will be allocated an individual trial identification number. All the data will be stored in a secure database, and the names and other personal information of subjects will not be recorded. People who have permission from the corresponding author can access this database. Moreover, researchers can email the corresponding author to have access rights to enable international prospective meta-analyses.

We have no plan for interim analysis until the study is achieved. We plan to conduct a subgroup analysis to explore the effect of treatment according to the following pre-specified subgroup: (1) unparturient and transparturient; (2) intraoperative circulatory stabilization and intraoperative circulatory instabilities.

## Discussion

HFNO has been widely applied in the clinic to improve oxygenation for acute hypoxemic respiratory failure [20, 21] and perioperative periods in recent years. Previous studies have demonstrated the efficacy of HFNO as an oxygenation, pre-oxygenation, and apneic oxygenation strategy in pregnant women [17]. Zhou et al. [22] confirmed that HFNO treatment at the rate of 50 L/min for 20 min did not increase the risk of aspiration in healthy fasted parturients breathing spontaneously. HFNO has also been used for pregnant women admitted to the intensive care unit with coronavirus disease 2019 as a ventilatory support method [23]. However, studies exploring the effects of maternal oxygen supplementation with HFNO on fetuses are lacking. Our study will provide new evidence in this aspect, and illustrate the effect the maternal oxygen inhalation as an intrauterine resuscitation intervention during elective cesarean section.

The intrapartum acid–base status of the fetus represents the neonatal condition just before birth, and UA blood gas analysis is the main basis for assessing fetal acidemia and estimating short- and long-term morbidity [24, 25]. Most researches used the UA pH as the primary outcome to assess the newborn status. But a descriptive study, including 2554 single deliveries, suggested that UA lactate may be a more correct indicator of fetal asphyxia at delivery than pH [26], and lactate is a direct end product of anaerobic metabolism [27, 28]. Data from animal models show that fetal lactate increases earlier in hypoxia, and persists longer than pH [29]. A large prospective cohort study also suggested that UA lactate is a more discriminating measure of neonatal morbidity at term than pH [28]. Consequently, in this research, UA lactate is chosen as the primary outcome.

Khaw et al. have concluded that hyperoxia may increase fetal oxidative stress and have a negative effect [6]. But the mean duration of oxygen administration was longer than 50 min. In another study by the same team, however, there were no differences in maternal or umbilical oxidative stress indices between the groups, and the mean oxygen exposure duration was 30 min [8]. Therefore, in our study, the duration of oxygen exposure is limited to the time between the completion of anesthesia placement and neonatal delivery. The time is usually 15–30 min in our center and rarely more than 40 min. In order to clarify the effect of hyperoxia on oxidative stress, malondialdehyde (MDA) of UA blood will also be measured.

In conclusion, for this research, we want to demonstrate whether supplemental oxygen with HFNO has a positive effect on fetal outcomes and decreases the incidence of fetal acidemia in in pregnant women who undergo elective cesarean section. The results of this research will be reported to peer-reviewed journals. Furthermore, we hope HFNO can be used for women with fetal distress to improve fetal outcomes, as an effective intrauterine resuscitation technique during emergency cesarean section.

## Trial status

This trial is ongoing and actively recruiting. The current version of the study protocol is version 3.0 and approved on 9 March 2023. Patient recruitment started on 1 July 2023 and is expected to be finished by 31 December 2023.

### Supplementary Information

**Additional file 1. **SPIRIT 2013 checklist.

## Data Availability

The datasets analyzed during the current study and statistical code are available on reasonable request through email to the corresponding author, as is the full protocol.

## Abbreviations

HFNO: High-flow nasal oxygen
UA: Umbilical artery
ASA: American society of anesthesiologists
BMI: Body mass index
CRF: Case report form
ECG: Electrocardiograph
HR: Heart rate
AEs: Adverse events

## References

[1] Moors S, Bullens LM, van Runnard Heimel PJ, Dieleman JP, Kulik W, Bakkeren DL (2020). The effect of intrauterine resuscitation by maternal hyperoxygenation on perinatal and maternal outcome: a randomized controlled trial. Am J Obstet Gynecol MFM.

[2] Reddy UM, Weiner SJ, Saade GR, Varner MW, Blackwell SC, Thorp JM (2021). Intrapartum Resuscitation Interventions for Category II Fetal Heart Rate Tracings and Improvement to Category I. Obstet Gynecol.

[3] Corke BC, Datta S, Ostheimer GW, Weiss JB, Alper MH (1982). Spinal anaesthesia for Caesarean section. The influence of hypotension on neonatal outcome. Anaesthesia..

[4] Maisonneuve E, Audibert F, Guilbaud L, Lathelize J, Jousse M, Pierre F (2011). Risk factors for severe neonatal acidosis. Obstet Gynecol.

[5] Gunaydin B, Nas T, Biri A, Koc E, Koc A, McCusker K (2011). Effects of maternal supplementary oxygen on the newborn for elective cesarean deliveries under spinal anesthesia. J Anesth.

[6] Khaw KS, Wang CC, Ngan Kee WD, Pang CP, Rogers MS (2002). Effects of high inspired oxygen fraction during elective caesarean section under spinal anaesthesia on maternal and fetal oxygenation and lipid peroxidation. Br J Anaesth.

[7] Ahuja V, Gombar S, Jaswal S, Kaur J, Gupta P, Chawla D (2018). Effect of maternal oxygen inhalation on foetal free radical activity: a prospective, randomized trial. Acta Anaesthesiol Scand.

[8] Khaw KS, Wang CC, Ngan Kee WD, Tam WH, Ng FF, Critchley LA (2009). Supplementary oxygen for emergency Caesarean section under regional anaesthesia. Br J Anaesth.

[9] Biswas J, Choudhury A, Das S, Mukhopadhyay P, Pal A, Jana D (2019). Analysis of Neonatal Outcome with Supplemental Oxygen to Mother during Elective Cesarean Section under Spinal Anesthesia: A Prospective Randomized Controlled Trial. Anesth Essays Res.

[10] Raghuraman N, Temming LA, Doering MM, Stoll CR, Palanisamy A, Stout MJ (2021). Maternal Oxygen Supplementation Compared With Room Air for Intrauterine Resuscitation: A Systematic Review and Meta-analysis. JAMA Pediatr.

[11] Siriussawakul A, Triyasunant N, Nimmannit A, Ngerncham S, Hirunkanokpan P, Luang-Aram S (2014). Effects of supplemental oxygen on maternal and neonatal oxygenation in elective cesarean section under spinal anesthesia: a randomized controlled trial. Biomed Res Int.

[12] Simon VB, Fong A, Nageotte MP (2018). Supplemental Oxygen Study: A Randomized Controlled Study on the Effect of Maternal Oxygen Supplementation during Planned Cesarean Delivery on Umbilical Cord Gases. Am J Perinatol.

[13] Chatmongkolchart S, Prathep S (2016). Supplemental oxygen for caesarean section during regional anaesthesia. Cochrane Database Syst Rev..

[14] Kelly MC, Fitzpatrick KT, Hill DA (1996). Respiratory effects of spinal anaesthesia for caesarean section. Anaesthesia.

[15] Merry AF, van Waart H, Allen SJ, Baker PA, Cumin D, Frampton CMA (2022). Ease and comfort of pre-oxygenation with high-flow nasal oxygen cannulae vs. facemask: a randomised controlled trial. Anaesthesia..

[16] Tan PCF, Millay OJ, Leeton L, Dennis AT (2019). High-flow humidified nasal preoxygenation in pregnant women: a prospective observational study. Br J Anaesth.

[17] Zhou S, Zhou Y, Cao X, Ni X, Du W, Xu Z (2021). The efficacy of high flow nasal oxygenation for maintaining maternal oxygenation during rapid sequence induction in pregnancy: a prospective randomised clinical trial. Eur J Anaesthesiol.

[18] Gaertner VD, Bassler D, Zimmermann R, Fontijn JR (2021). Reference Values for umbilical artery lactate by mode of delivery and gestational age: a retrospective observational study. Neonatology.

[19] Hamel MS, Anderson BL, Rouse DJ (2014). Oxygen for intrauterine resuscitation: of unproved benefit and potentially harmful. Am J Obstet Gynecol.

[20] Rochwerg B, Einav S, Chaudhuri D, Mancebo J, Mauri T, Helviz Y (2020). The role for high flow nasal cannula as a respiratory support strategy in adults: a clinical practice guideline. Intensive Care Med.

[21] Grieco DL, Maggiore SM, Roca O, Spinelli E, Patel BK, Thille AW (2021). Non-invasive ventilatory support and high-flow nasal oxygen as first-line treatment of acute hypoxemic respiratory failure and ARDS. Intensive Care Med.

[22] Zhou S, Cao X, Zhou Y, Xu Z, Liu Z (2023). Ultrasound Assessment of Gastric Volume in Parturients After High-Flow Nasal Oxygen Therapy. Anesth Analg.

[23] Péju E, Belicard F, Silva S, Hraiech S, Painvin B, Kamel T (2022). Management and outcomes of pregnant women admitted to intensive care unit for severe pneumonia related to SARS-CoV-2 infection: the multicenter and international COVIDPREG study. Intensive Care Med.

[24] Malin GL, Morris RK, Khan KS (2010). Strength of association between umbilical cord pH and perinatal and long term outcomes: systematic review and meta-analysis. BMJ (Clinical research ed).

[25] Thorp JA, Rushing RS (1999). Umbilical cord blood gas analysis. Obstet Gynecol Clin North Am.

[26] Gjerris AC, Staer-Jensen J, Jørgensen JS, Bergholt T, Nickelsen C (2008). Umbilical cord blood lactate: a valuable tool in the assessment of fetal metabolic acidosis. Eur J Obstet Gynecol Reprod Biol.

[27] Allanson ER, Waqar T, White C, Tunçalp Ö, Dickinson JE (2017). Umbilical lactate as a measure of acidosis and predictor of neonatal risk: a systematic review. BJOG.

[28] Tuuli MG, Stout MJ, Shanks A, Odibo AO, Macones GA, Cahill AG (2014). Umbilical cord arterial lactate compared with pH for predicting neonatal morbidity at term. Obstet Gynecol.

[29] Engidawork E, Chen Y, Dell'Anna E, Goiny M, Lubec G, Ungerstedt U (1997). Effect of perinatal asphyxia on systemic and intracerebral pH and glycolysis metabolism in the rat. Exp Neurol.

